# Outcomes of hypofractionated stereotactic body radiotherapy boost for intermediate and high-risk prostate cancer

**DOI:** 10.1186/s13014-016-0585-y

**Published:** 2016-01-21

**Authors:** Mekhail Anwar, Vivian Weinberg, Zachary Seymour, I. Joe Hsu, Mack Roach, Alex R. Gottschalk

**Affiliations:** Department of Radiation Oncology, University of California San Francisco, 1825 4th Street, San Francisco, CA 94158 USA; Department of Radiation Oncology, University of California San Francisco, 1600 Divisadero St. Suite H1031, San Francisco, CA 94143-1708 USA; Department of Radiation Oncology, Beaumont Health, 44201 Dequindre Rd, Troy, MI 48085 USA; Department of Radiation Oncology and Urology, University of California San Francisco, 1600 Divisadero St. Suite H1031, San Francisco, CA 94143-1708 USA

**Keywords:** Stereotactic body radiotherapy, Boost, Prostate cancer

## Abstract

**Background and purpose:**

Treatment of intermediate and high-risk prostate cancer with a high BED has been shown to increase recurrence free survival (RFS). While high dose rate (HDR) brachytherapy, given as a boost is effective in delivering a high BED, many patients are not candidates for the procedure or wish to avoid an invasive procedure. We evaluated the use of stereotactic body radiotherapy (SBRT) as a boost, with dosimetry modeled after HDR-boost.

**Material and methods:**

Fifty patients were treated with two fractions of SBRT (9.5-10.5 Gy/fraction) after 45 Gy external-beam radiotherapy, with 48 eligible for analysis at a median follow-up of 42.7 months.

**Results:**

The Kaplan-Meier estimates of biochemical control post-radiation therapy (95 % Confidence Interval) at 3, 4 and 5 years were 95 % (81–99 %), 90 % (72–97 %) and 90 % (72–97 %), respectively (not counting 2 patients with a PSA bounce as failures). RFS (defined as disease recurrence or death) estimates at 3, 4 and 5 years were 92 % (77–97 %), 88 % (69–95 %) and 83 % (62–93 %) if patients with PSA bounces are not counted as failures, and were 90 % (75–96 %), 85 % (67–94 %) and 75 % (53–88 %) if they were. The median time to PSA nadir was 26.2 months (range 5.8–82.9 months), with a median PSA nadir of 0.05 ng/mL (range <0.01–1.99 ng/mL). 2 patients had a “benign PSA bounce”, and 4 patients recurred with radiographic evidence of recurrence beyond the RT fields. Treatment was well tolerated with no acute G3 or higher GI or GU toxicity and only a single G3 late GU toxicity of urinary obstruction.

**Conclusions:**

SBRT boost is well-tolerated for intermediate and high-risk prostate cancer patients with good biochemical outcomes and low toxicity.

## Introduction

Patients with intermediate and high-risk prostate cancer have poorer clinical outcomes than patients with low-risk prostate cancer, and in need of more intensified therapeutic options. Although the role of hormone therapy (HT) [1] in increasing recurrence free survival (RFS) and overall survival (OS) has been well established, the optimum dose and fractionation of radiation is still being investigated. Multiple randomized trials have demonstrated an increased RFS with dose escalation [2–4], albeit no difference in OS. Coupled with a potentially low alpha-beta ratio [5, 6] for prostate adenocarcinoma, recent strategies for improving clinical outcomes have focused on hypofractionated radiotherapy to deliver a higher bioequivalent dose (BED) over conventionally fractionated (1.8-2 Gy/fraction) external beam radiotherapy (CF-EBRT), as well as shortening treatment regimens for increased cost-effectiveness [7]. These efforts led to use of high dose rate brachytherapy (HDR), both as a boost [8–10] and as monotherapy [11–14]. Further supporting the use of hypofractionated radiotherapy, a lower PSA nadir has been associated with increased freedom from biochemical failure [15–18] in prostate cancer, and the results of studies employing HDR demonstrate PSA nadirs in the range of 0.1 ng/mL [9], lower than the 0.5 ng/mL level typically associated with CF-EBRT. Unfortunately, HDR requires an invasive operative procedure and many prostate cancer patients are often not candidates due to their age, co-morbidities, or preference not to have surgery. Therefore, our goal was to develop and demonstrate a non-invasive method of delivering a dose and fractionation equivalent to an HDR boost using stereotactic body radiotherapy (SBRT) for patients with intermediate and high-risk prostate cancer.

While SBRT has a long history in the treatment of CNS and thoracic malignancies, several recent studies have demonstrated its feasibility and applicability for treatment of low and intermediate risk prostate cancer as monotherapy [19–21] with minimal toxicity [22], but few studies have rigorously examined SBRT in the boost setting. Our hypothesis was that the SBRT delivered in 2 fractions of 9.5 to 10.5 Gy should result in equivalent biochemical control and toxicity profile as HDR without the need for an invasive, operative procedure. Dosimetry was modeled after RTOG 0321, replicating the rapid delivery of high doses, as well as the ability to achieve tight conformality, sparing nearby normal tissues. This study differs from previous studies in that we present the results of SBRT as a boost after CF-EBRT in a higher risk group of patients and discuss PSA kinetics, biochemical control, toxicity and patterns of recurrence.

## Patients and methods

### Ethics, consent, and permissions

Patients with intermediate and high-risk prostate cancer treated with SBRT as a boost from August 2006 to August 2012 were followed with approval from the XXXX Committee on Human Research.

### Patient selection

Patients with biopsy proven prostate adenocarcinoma were seen in a multidisciplinary clinic and counseled on treatment options, including surgery and radiotherapy. Patients were eligible for this study if they were intermediate (Gleason 3 + 4 or 4 + 3) or high (Gleason ≥8) grade, node negative, without metastases, and treated with SBRT as a boost to the prostate after a course of CF-EBRT to the prostate and seminal vesicles (with or without whole pelvic radiation). Of the 50 eligible patients, 48 patients met these criteria and 2 were excluded due to no available PSA follow-up. T-stage ranged from T1c to T3b and pre-treatment PSA arranged from 3.6 to 150 ng/mL. Patients were ≥18, with median age of 70 (range 47.1–85.2) years. Patients were eligible for treatment with or without hormone therapy (HT).

### Treatment

Of the 48 eligible patients, 45 had information regarding HT available. The majority (42) of these patients received HT, consisting of 2 months of neoadjuvant HT with Lupron and Casodex or Firmagon, followed by 5 weeks of HT. Thirty of these patients were subsequently treated with >3 additional months of adjuvant HT, with a goal of 4–6 months of HT for all intermediate risk patients, and 2 years of HT for high risk patients [23]. HT was discontinued early if the patient experienced side effects significantly affecting their quality of life. The two SBRT fractions were delivered either consecutively or every-other-day [24].

### Radiation technique

The specifics of the SBRT technique have been described previously [25], but briefly, the dose and fractionation are based on the XXX HDR boost experience [26], with 9.5 Gy or 10.5 Gy in 2 fractions prescribed to an isodose of 60-80 %. This corresponds to a biological equivalent dose (BED) of 278 to 336 with an alpha/beta of 1.5 or a BED of 158 to 189 with an alpha/beta of 3. A 2 mm planning treatment volume (PTV) expansion is used, except posteriorly, where the prostate abuts the rectum, the expansion is 0 mm. The posterior expansion is reduced to mitigate rectal toxicity. Patients were initially treated with external beam radiotherapy to the whole pelvis, extending to between L4/L5 and L5/S1 as the superior border, and including the internal and external iliacs and obdurator nodes to 45 Gy in 25 fractions. The prostate and seminal vesicles were treated concurrently to 45–50 Gy using a simultaneous integrated boost (SIB) to the prostate and seminal vesicles of 2 Gy per fraction. The whole pelvis was treated if the risk of pelvic nodal involvement was >15 % by the Roach equations [27]. Aside from the use of an SIB, radiotherapy planning for the conventionally fractionated portion was done as in RTOG 0321 [8]. Because gold seed fiducials are used for daily alignment, the PTV margin did not exceed 1 cm. Prior to radiation treatment, 3 fiducial markers were inserted into the prostate, enabling real-time tracking of and automatic beam adjustment for inter- and intra-fraction prostate motion, for whole pelvic RT and SBRT, respectively. Imaging for SBRT was taken once every 60 s.

Treatment planning was completed with dosimetric constraints similar to those reported by Fuller [28] for inverse-planned HDR brachytherapy with PTV V100% >95 %, D0.1 ml < V120 to the urethra, and rectum and bladder V75% < 1 mL. In cases were rectum and bladder doses were not achievable, based on the prostatic interface, doses to rectum and bladder were allowed up to V75% of 5 mL [29].

### Follow-up

Patients were evaluated every 3 months for 2 years, and then every 6 months up to at least 5 years, and annually thereafter with PSA and testosterone testing. PSA results below the detection limit of the assay were entered as the respective detection limit (for example 0.1 ng/mL for a value of <0.1 ng/mL) for data analysis as being the most conservative estimate. Testosterone testing was discontinued once it returned into the normal range. Toxicity was reviewed according to the CTCAE v4.0 scale.

### PSA kinetics: nadir, bounce and failure

PSA nadir was determined for patients with sufficient PSA follow-up, determined by at least 2 PSA measurements after completion of HT or at least 2 PSA measurements for patients not undergoing HT. Of the 48 evaluable patients, 43 patients met these criteria: treatment with HT was unknown for 3 patients, and 2 patients only had 1 post-HT PSA measurement.

Patients were counted as biochemical failures according to the Phoenix definition [30] (PSA >2 ng/mL above the currently observed PSA nadir). PSA bounce, a benign phenomenon frequently noted after radiotherapy, was defined as an increase in PSA above the currently observed nadir greater than 2 ng/ml, with a subsequent decline in PSA without further treatment. Outcomes were also computed by counting PSA bounces as failures with the date of biochemical failure the date of the increase of at least 2 ng/mL above the PSA nadir and also repeated counting them as benign events.

### Statistical analysis

A single cohort of patients was uniformly treated with CF-EBRT, SBRT and, for almost all, with HT and then followed prospectively. Descriptive statistics (e.g., means, medians, proportions) were calculated to characterize patient, disease and treatment features. The Kaplan-Meier method was used to estimate the probability of biochemical control and of RFS with both measured from the end of RT. Biochemical failure was defined strictly according to the Phoenix definition and repeated adjusting for a PSA bounce. RFS failure was the first event of either any disease recurrence or death. Disease recurrence is defined as biochemical (PSA) failure per the Phoenix definition.

## Results

### Patient characteristics

Of the 48 evaluable patients (Table 1), the median follow-up was 42.7 months (range 5.3–82.9 months). The median number of PSA measurements during follow-up was 8 (range 1–24), with a median of 7 (range 1–19) PSA measurements post-HT for those treated with any HT. The median pre-treatment PSA was 10.0 ng/mL (range 3.6–150 ng/mL), and 8 of 48 (17 %) had PSA ≥20 ng/mL. 27 patients (56 %) were Gleason 7, with 15 patients Gleason 3 + 4 and 12 patients Gleason 4 + 3. Twenty-one patients (44 %) had tumors that were scored as Gleason 8–10. A significant number (42 %) of patients had either extracapsular extension (T3a) or seminal vesicle invasion (T3b) on biopsy or ultrasound. The remainder of patients were T1c (25 %), T2a (27 %), and T2b (6 %). All patients underwent conventionally fractionated external beam radiotherapy and the majority (88 %) received hormone therapy, with 31 % receiving >6 months of HT. 48 % of patients received a boost of 9.5 Gyx2 fractions, while 52 % received 10.5 Gyx2 fractions.

**Table 1:** Baseline patient and treatment characteristics: (n = 48)

Median Age (range)	70 years (47.1 – 85.2)
Median Pre-Treatment PSA (range)	10.0 ng/mL (3.6 – 150.0)
≥20 ng/mL	8 (17 %)
Gleason Score	
7	27 (56 %)
3 + 4	15 (31 %)
4 + 3	12 (25 %)
8-10	21 (44 %)
T Stage	
T1c	12 (25 %)
T2a	13 (27 %)
T2b	3 (6 %)
T3a,b	20 (42 %)
# High Risk^a^	34 (71 %)
Median Duration HT (range)	6 months (0 – 28)
None	3 (6 %)
3 months	12 (25 %)
4–6 months	15 (31 %)
7–12 months	8 (17 %)
24–28 months	7 (15 %)
Unknown	3 (6 %)
CTV Dose	
19 Gy	23 (48 %)
21 Gy	25 (52 %)
Median Follow-up (range)	42.7 months (5.3 – 82.9)
# PSA Follow-up Measurements	
Median (range)	8.5 (1 – 24)
Median Post HT (range)	7 (1 – 19)

^a^High Risk is defined as pretreatment ≥20 ng/mL, GS 8–10 or T stage 3

### Biochemical control

Of the 48 evaluable patients, 6 patients had a PSA rise ≥2 ng/mL above the current nadir, but two of these patients subsequently had a decline in PSA and which was then determined to be a PSA bounce. Follow-up is too short to estimate overall survival with only 2 observed deaths as of this analysis, without either patient showing any evidence of disease.

The Kaplan-Meier estimates of biochemical no evidence of disease (bNED) with 95 % confidence intervals are 95 % (81–99 %) at 3 years, 90 % (72–97 %) at 4 years, and 90 % (72–97 %) at 5 years. If PSA bounces are counted as failures, then the estimates of bNED are 93 % (78–98 %) at 3 years, 88 % (69–96 %) at 4 years, and 82 % (61–93 %) at 5 years.

### Recurrence free survival

The Kaplan-Meier estimates of RFS estimates at 3, 4 and 5 years were 92 % (77–97 %), 88 % (69–95 %) and 83 % (62–93 %) if patients with PSA bounces are not counted as failures, and 90 % (75–96 %), 85 % (67–94 %) and 75 % (53–88 %) if they were.

### PSA nadir

A lower PSA nadir is associated with improved clinical outcomes, and therefore we evaluated both the PSA nadir and time to nadir. For 43 patients with sufficient PSA follow-up to evaluate a PSA nadir, defined as having at least 2 PSA measurements without any HT or after completing HT, the median time to nadir was 26.2 months (range: 5.8–82.9 months), and the median PSA nadir was 0.05 ng/mL (range <0.01–1.99 ng/mL). The majority of these patients (72 %), had a PSA nadir ≤0.10 ng/mL, and only 2 patients (5 %) had a PSA nadir >1 ng/mL and both of these patients had not received any HT. These estimates of PSA nadir and time to nadir do not change if PSA bounces are not counted as failures because the values change for only 1 of the 2 patients with a PSA bounce.

The use of hormone therapy had a noticeable effect on PSA nadir: the 3 patients who did not receive any HT had the highest PSA nadir for the study cohort (0.97, 1.51 and 1.99 ng/mL).

### PSA bounce

For the 2 patients who experienced a benign PSA bounce, initial failure by the Phoenix definition occurred at 50 and 11 months, while the duration for bNED was 54 and 58, months, respectively, when considering the bounce as benign. The initial PSA nadirs for these two patients were 0.46 and 0.55, and the maximum PSA reflecting the bounce, 4.9, and 5.54 ng/ml, respectively. These values subsequently declined to 1.3, and 0.04 ng/ml, respectively, at last follow-up.

### Patterns of failure

Of the 4 patients who recurred biochemically (without PSA bounce), all recurred outside the radiation field, including 2 with peri-aortic nodal involvement, 33 and 21 months after completion of SBRT. These nodes were discovered on imaging 3 and 9 months, respectively, after biochemical failure. The third patient developed a bone metastasis at T2 79 months after radiation. A fourth patient, with T3a Gleason 4 + 5 disease, developed metastatic disease to the bone discovered on PET/CT 52 months after SBRT and 4 months after biochemical failure.

### Toxicity

No patient in this study had acute Grade 3 or higher genitourinary (GU) or gastrointestinal (GI) toxicity (Table 2). Twenty-three (48 %) and 18 (37 %) patients had an acute grade 1 (G1) and G2 GU toxicity, while only 20 (42 %) and 5 (10 %) patients had an acute G1 and G2 GI toxicity, respectively. A single case of late G3 GU toxicity occurred with urinary obstruction, but resolved. 10 and 12 patients had late G1 and 2 GU toxicity, respectively, and the majority (82 %) of these patients had acute G1 or 2 GU toxicity as well. Late GI toxicity was rare, with only 6 patients (12.5 %) having late grade 1 toxicity, and only 2 of these patients had acute GI toxicity. There was no G2 or higher late GI toxicity.

**Table 2:** Frequency of acute and late Genitourinary (GU) and Gastrointestinal (GI) toxicity

		# Resolved
Acute GU:		
Grade 0	7 (15 %)	-
Grade 1	23 (48 %)	16
Grade 2	18 (37 %)	17 (1 dose de-escalation)
Acute GI:		
Grade 0	23 (48 %)	
Grade 1	20 (42 %)	10
Grade 2	5 (10 %)	1
Late GU:		
Grade 0	25 (52 %)	-
Grade 1	10 (21 %)	3
Grade 2	12 (25 %)	9
Grade 3	1 (2 %)	1 (Obstruction)
Late GI:		
Grade 0	42 (87.5 %)	-
Grade 1	6 (12.5 %)	1

Acute Toxicity: Maximum grade observed during the first 6 months from start of protocol therapy

## Discussion

The patients studied here represent an older, higher risk cohort, often typical of patients who have advanced prostate cancer and are not surgical candidates. The median age was 70 and 71 % of patients were high-risk, as defined by D’Amico et al. [23]. Notably, almost half of patients (44 %) had Gleason 8 disease or higher, or extension outside of the prostate (42 % T3a/b), with 67 % of patients having one or both features. Consistent with the proven role of hormone therapy in increasing overall survival in high-risk patients, 88 % received hormone therapy.

The results presented here compare favorably with historical series studying intermediate and high-risk patients using CF-EBRT. In RTOG 8610 [31] only 40 % of patients were bNED at 5 years, while RTOG 9202 [32] and EORTC 22863 [33] found a 5 year disease free survival rate of 46.4 and 76 %, respectively. With the caveat that these studies contained a different patient population, occurred pre-dose escalation, and defined biochemical failure in different ways, our 5 year estimates of bNED of 90 % and RFS of 83 %, compare favorably to the these trials.

The high rate of biochemical control for this relatively high-risk cohort may also be due to the incorporation of a hypofractionated boost, leveraging the low α/β ratio of prostate cancer. The possibility of a low α/β ratio in prostate cancer has prompted clinical investigations utilizing a hypofractionated boost delivered with HDR, providing a more appropriate comparison for the results presented here. The Seattle Prostate Institute reported a 15 year biochemical control rate of 80 and 68 % for intermediate and high-risk groups, respectively [34], treated with a permanent seed implant after pelvic radiotherapy. Another randomized study comparing HDR boost of 8 Gyx2 fractions versus CF-EBRT showed PSA relapse-free survival of 97 and 96 % for intermediate and high-risk patients, respectively [35]. At UCSF, 165 patients treated with a HDR boost (9.5 Gyx2 fractions or 6 Gyx3 fractions) [36] showed PSA control rates of 87 and 93 % for the two dose subsets, respectively, at 5 years. A dose escalation study [37] showed BED > 268 Gy (α/β = 1.2) delivered with HDR yielded a 10 year biochemical control rate of 81 %. A combined analysis of HDR boost patients [38] showed that biochemical control at 5 years was 88 and 69 % for patients with one and two risk factors (stage ≥ T2b, GS ≥ 7, and PSA ≥ 10 ng/mL), respectively. Therefore, the results presented here indicate comparable biochemical outcomes to recent HDR-boost series that employed a similar dose and fractionation.

The results presented here also compare favorably with recent SBRT series in prostate cancer, with regards to both biochemical control and toxicity (Table 3). A multi-institutional analysis [19] of localized prostate cancer treated with SBRT, with a median follow-up of 36 months, found the 5-year biochemical relapse free survival rate was 84 and 81 % for intermediate and high-risk prostate cancer patients, respectively. Similar to the patients studied here, Katz [39] reported outcomes using SBRT as a boost for intermediate and high-risk patients, finding 3-year biochemical control rates of 89.5 and 77.7 %, respectively. Treatment was well tolerated, with only 6.8 % acute G2 GU toxicity and 6.7 % G2 rectal toxicity. Albeit with a follow-up of only 33 months, the long-term toxicity results are encouraging, with a rate of 91.8 % for G2 or higher rectal toxicity-free survival. Future efforts at assessing toxicity should include patient reported outcomes.

**Table 3:** Comparison of outcomes of contemporary SBRT

Author (Year)	N	Pt type	Dose	FU (yr)	Toxicity	Outcome	Ref
King (2009)	67	Low-risk; PSA < 10, G < = 3 + 4; <= T2a/b	7.25 x 5	2.7	GU: Grade 4 (0 %) 3 (3 %), 2 (5 %), and 1 (23 %) respectively. Rectal Grade 3 (0 %), 2 (2 %), and 1 (12.5 %).	(5 years) 94 % relapse free survival	[24]
Boike (2011)	45	PSA ≤ 20, G ≤ 7, ≤T2b	9-10 x 5	2.5	GI: 3 (2 %), 2 (16 %); GU: 3 (4 %); 2 (27 %)		[48]
Chen (2013)	100	Low/intermediate risk (8 high)	7-7.25 x 5	2.3	GU ≥2 (31 %); GI ≥ 2 (1 %)	(2 years) 99 % (31 % bounce, 21 % late transient urinary symptom flare)	[21]
Katz (2010)	73	Intermediate/High Risk	6-7 x 3 (Boost)	2.75	2 (7 %)	(3 years) 89.5 % (Intermediate); 77.7 % (high)	[39]
Suy (2010)	24		6.5 x 3 (Boost)	0.8	GU 2 (13 %); GI 2 (4 %)		[49]
Katz (2010)	304	Low/intermediate/high	7-7.25 x 5	2.5	Acute GU 2 (4.7 %); GI 2 (4 %); Late GU 3 (0.5 %); 2 (6 %) GI 2 (3 %)	4 failures (16 % bounce)	[50]
Jabarri (2012)	38	Low/intermediate/high	9.5 x 4 (mono); 9.5 x 2 (boost)	1	Acute GU 2 (42 %); GI 2 (11 %) Late G3 3 (5 %)	No failures	[25]
King (2013)	1100 (pooled)	Low (58 %), intermediate (30 %) and high-risk (11 %).	36.25 Gy in 4–5 fx	3		(5 years) 93 % (16 % bounce)	[19]

Although long-term results are needed to definitively establish the efficacy of SBRT as a boost modality in these patients, the recent introduction of SBRT into the clinic limits the available follow-up. However, the PSA nadir provides insight into the long-term biochemical control rate [40], with lower PSA nadir associated with increased freedom from biochemical failure [15–18]. In a matched-pair analysis [41], the PSA nadir from EBRT + HDR was 0.4 ng/ml, significantly lower than the 1.1 ng/ml with EBRT alone. Similarly, the University of Berlin [42] showed that 53 % of patients treated with HDR boost reached a PSA nadir of ≤0.5 ng/ml and the William Beaumont Hospital [43] reported that 70 % of patients had a PSA nadir of <0.5 ng/mL. Our results using SBRT compare favorably with these, with a PSA nadir of 0.05 ng/mL and a median time to nadir of 26 months. Of note, the PSA nadir continues to decline with longer follow-up. In a subset of these patients, we previously reported a median PSA nadir of 0.1 ng/mL at a median follow-up of 33.4 months [44]. When analyzing the PSA kinetics of SBRT monotherapy, PSA nadir also declines with longer follow-up [45]. Additional follow-up may yield an even lower median PSA nadir for this study cohort.

The toxicity reported here is similar to other series using EBRT with HDR and SBRT. RTOG 0321 used an HDR boost of 9.5 Gyx2 and noted 2.5 % late G3 and greater GU and GI toxicities. An updated analysis showed that 28 of 121 evaluable cases have grade 2+ GU toxicity [46], with V120 and greater associated with increased urethral toxicity. To mitigate this, the urethra is contoured on the co-registered MRI to implement urethral sparing. Using HDR accepted dose tolerances, only a single G3 urinary toxicity was observed. A recent summary of over 1000 patients undergoing SBRT monotherapy reported only minimal decline in quality of life measures after treatment, with reported grade 3 toxicity ranging between 1 and 3 % [47].

There are several limitations to this study. Although this is a relatively high-risk, older group of patients, it includes both intermediate and high-risk patients. Although testosterone was measured until it returned to the normal range, the use of HT and the slow recovery of testosterone may reduce the PSA value. Furthermore, no regular imaging of the prostate or re-biopsy was done, and therefore local control rates are only based on lack of PSA failure, and local recurrences cannot be categorically excluded. As with all non-randomized studies, patient selection can introduce bias, but this often results in patients with more adverse features being included in the study population due to the inclusion of older, non-operable candidates. Although the median follow-up is about 4 years, longer follow-up is needed to definitively assess the efficacy of SBRT in the boost setting.

## Conclusion

Hypofractionated SBRT is a feasible method to deliver the boost dose in an older, high-risk cohort, as demonstrated here. Although longer follow-up is needed, the preliminary biochemical control is similar to treatment with an HDR boost and to date, in patients with biochemical failure, there are no radiographically documented local failures. The low median PSA nadir of 0.05 ng/mL following SBRT is an improvement over standard EBRT and comparable to brachytherapy, adding to the evidence that prostate cancer has a low α/β and benefits from hypofractionated treatment. SBRT is a viable option to deliver the boost dose, achieving promising biochemical control and reduced PSA nadir with minimal toxicity without a surgical procedure.

### Ethics approval and consent to participate

This study was done in accordance with the Declaration of Helsinki, and approved by the University of California Committee on Human Research, #10-03010.

### Consent for publication

Not applicable.

## Abbreviations

CF-EBRT: Conventionally fractionated external beam radiotherapy
DFS: Disease-free survival
EBRT: External beam radiotherapy
HDR: High dose rate brachytherapy
SBRT: Stereotactic body radiotherapy
WPRT: Whole pelvic radiotherapy
